# IL-17 down-regulates the immunosuppressive capacity of olfactory ecto-mesenchymal stem cells in murine collagen-induced arthritis

**DOI:** 10.18632/oncotarget.10261

**Published:** 2016-06-23

**Authors:** Jie Tian, Ke Rui, Xinyi Tang, Wenxin Wang, Jie Ma, Xinyu Tian, Yungang Wang, Huaxi Xu, Liwei Lu, Shengjun Wang

**Affiliations:** ^1^ Department of Laboratory Medicine, The Affiliated People's Hospital, Jiangsu University, Zhenjiang, China; ^2^ Institute of Laboratory Medicine, Jiangsu Key Laboratory for Laboratory Medicine, Jiangsu University, Zhenjiang, China; ^3^ Department of Pathology and Centre of Infection and Immunology, The University of Hong Kong, Hong Kong, China

**Keywords:** olfactory ecto-mesenchymal stem cells, IL-17, suppressive capacity, Treg, collagen-induced arthritis, Immunology and Microbiology Section, Immune response, Immunity

## Abstract

Olfactory ecto-mesenchymal stem cells (OE-MSCs) are a population of cells which has been recognized as a new resident stem cell type in the olfactory lamina propria. OE-MSCs have been shown to exert their immunosuppressive capacity by modulating T cell responses, including up-regulation of regulatory T cells (Tregs) and down-regulation of Th1/Th17 cells. As an inflammatory cytokine, IL-17 plays a critical role in orchestrating the inflammatory response during the development of collagen-induced arthritis (CIA). However, it is unclear whether the increased level of IL-17 may affect the immunosuppressive function of OE-MSCs under inflammatory condition. In this study, we found that IL-17 could significantly reduce the suppressive capacity of OE-MSCs on CD4+ T cells and down-regulate the suppressive factors produced by OE-MSCs. Notably, IL-17 treatment abolished the capacity of OE-MSCs in inducing Treg expansion. In addition, knockdown of IL-17R in OE-MSCs significantly enhanced their therapeutic effect in ameliorating CIA upon adoptive transfer. Moreover, IL-17R knockdown-OE-MSCs could efficiently induce Tregs expansion and reduce Th1 and Th17 responses. Taken together, all these data suggest that IL-17R knockdown in OE-MSCs may provide a novel strategy in maintaining their immunosuppressive properties for the treatment of autoimmune diseases.

## INTRODUCTION

Rheumatoid arthritis (RA) is an autoimmune disease caused by chronic joint inflammation, leading to cartilage destruction and bone erosion [1]. Although the precise etiology of RA is still elusive, various proinflammatory cytokines and autoreactive T cells are essential elements in its pathogenesis. The activation of Th1 cells and Th17 cells has been considered to be critical factors in the pathogenisis of cell-mediated autoimmune arthritis [2, 3]. In contrast, Treg cells and Th2 cells are protective in RA and in animal models of collagen-induced arthritis (CIA) [4, 5]. The development of therapies in RA treatment progressed slowly. Currently, although products such as IL-1 antagonists, TNF inhibitors and anti-IL-6 receptor antibody are effective in RA treatment, not all the RA patients respond to these cytokine associated products, and none of these drugs are curative for RA [6]. Thereafter, novel strategies for RA treatment should be explored. Mesenchymal stem cells (MSCs) are multipotent progenitor cells, which can be isolated from bone marrow, cord blood, muscle and fat tissue. MSCs have the capability to differentiate into adipogenic, osteogenic, and chondrogenic lineages, and have the ability to differentiate to lineages of mesenchymal tissues, including bone, cartilage and adipose tissues [7]. Moreover, MSCs have been considered to have potent immunosuppressive and anti-inflammatory effects *via* cell-cell contact or by secreting soluble factors, such as IL-10, NO, TGF-β, indoleamine 2, 3-dioxygenase (IDO), prostaglandin E2 (PGE2) and so on [8, 9]. They effectively impair the proliferation or the activation of T cells, B cells, NK cells and antigen presenting cells, thus raising great interest for their potential therapeutic application. Accumulating experimental and clinical evidence has demonstrated that MSCs could lead to significant immunosuppressive effects when treating different inflammatory and autoimmune diseases [10, 11]. Recently, olfactory ecto-mesenchymal stem cells (OE-MSCs) have been recognized to be a new resident stem cell type in the olfactory lamina propria. OE-MSCs sited in nasal cavity, developing primarily from neural crest cells, possessing high proliferation rate, self-renewal capability and multiple differentiation capability. Our previous work has demonstrated that OE-MSCs can exert their immunosuppressive capacity in modulating T cell responses and ameliorate disease severity in CIA mice [12]. Although the MSC-based immunotherapy has shown significant effect in CIA treatment, the application of MSCs in clinic still encounters different difficulties, such as some patients with autoimmune diseases are not sensitive to the MSCs treatment.

IL-17 has recently been implicated in the pathogenesis of various autoimmune diseases, including RA and the mouse model CIA. High level of IL-17 was found in the serum and synovial fluid of RA patients [13–15]. Accumulating evidence has suggested that IL-17 is an essential pathogenic cytokine that is associated with autoimmune joint inflammation. Furthermore, IL-17 is reported to be one of the critical reasons leading to the failure of MSC-based immunotherapy, such as mouse colitis [15].

Although it is well known that OE-MSCs possess immunosuppressive effect, it is unclear whether IL-17 will have negative regulation of OE-MSCs and then affect the effect of MSCs application, especially in diseases with high levels of IL-17 or the patients in the stage of high concentration of IL-17. To investigate it, we used IL-17 to stimulate OE-MSCs and found that IL-17 could significantly reduce the suppressive effect of OE-MSCs, and IL-17 treated OE-MSCs lost the capacity of promoting the expansion of Tregs. In addition, the IL-17R knockdown-OE-MSCs showed more efficient effect in treating CIA mouse when compared to the control OE-MSCs, which suggests that at the peak of IL-17 during the CIA development, OE-MSCs might be regulated and then cause the failure of treatment. Thereafter, blocking the IL-17/IL-17R pathway might be an effective strategy favors the OE-MSC clinical application.

## RESULTS

### IL-17 down-regulates the suppressive capacity of OE-MSCs

**Figure 1:**
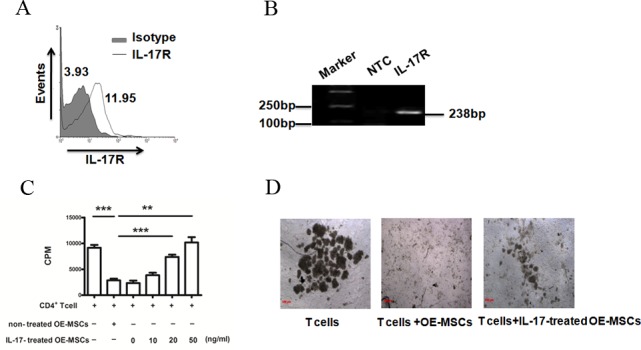
IL-17 reduces the suppressive capacity of OE-MSCs **A.** OE-MSCs were stained for IL-17R with anti-IL-17R antibody (thick line histogram) or rat IgG2a (solid gray histogram) and then analyzed using flow cytometry. **B.** RNA isolated from OE-MSCs was subjected to RT-PCR to measure IL-17R mRNA expression. **C.**, **D.** OE-MSCs were stimulated with or without IL-17 (0, 10, 20, 50 ng/ml) for 48 h, and then cells were harvested to co-cultured with CD4^+^ T cells in the presence of anti-CD3 mAb and anti-CD28 mAb for 72 h. Suppression of T-cell proliferation was measured by [^3^H]-thymidine incorporation (C), and the status of cells in the co-culture system was also observed under the microscope (100×, D). Data are presented as mean ± SD pooled from three independent experiments. ***p < 0.001, **p < 0.01.

The OE-MSCs were successfully isolated from nasal cavity of mice and cultured as described in Materials and Methods. Flow cytometric analysis showed that OE-MSCs expressed CD29, CD44, CD90 but not CD34, CD45, CD11b markers (Supplementary Figure 1A). In addition, OE-MSCs could differentiate into osteocytes and adipocytes (Supplementary Figure 1B), which suggests that OE-MSCs have similar phenotypic features and multiple-lineage differentiation capacities of MSCs. Next, to investigate the effect of IL-17 on OE-MSCs, we first examined whether OE-MSCs express IL-17R. Both flow cytometry analysis and RT-PCR showed that the OE-MSCs expressed IL-17R (Figure 1A, 1B). IL-17 is a pro-inflammatory cytokine whose level is increased during autoimmune diseases. In an effort to determine if IL-17 treatment has any effect on the suppressive function of OE-MSCs *in vitro*, we co-cultured CD4^+^ T cells with OE-MSCs treated with or without IL-17. As shown in Figure 1C, compared to the non-treated OE-MSCs group, the suppression of OE-MSCs on CD4^+^ T cells was blocked in the IL-17-treated OE-MSCs group, and the status of cells in the co-culture system was also observed under the microscope (100×, Figure 1D), which suggests that the suppressive activity of OE-MSCs on CD4^+^ T cells was significantly inhibited by IL-17 stimulation.

### Suppressive factors expressed by OE-MSCs are down-regulated upon IL-17 treatment

**Figure 2:**
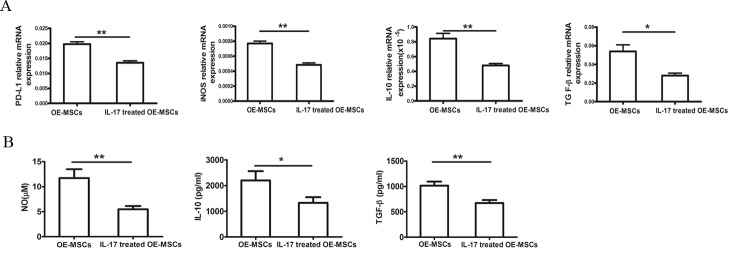
IL-17 down-regulates the suppressive factors of OE-MSCs **A.**, **B.** OE-MSCs were stimulated with or without IL-17 (20 ng/ml) for 48 h, and then cells were harvested to co-culture with CD4^+^ T cells in the presence of anti-CD3 mAb and anti-CD28 mAb for 72 h, (A) and then OE-MSCs were collected to extract total RNA, and qRT-PCR was used to analyze the mRNA expression of PD-L1, iNOS, IL-10 and TGF-β, (B) and the supernatant was harvested to detect NO, IL-10 and TGF-β. Data are presented as mean ± SD pooled from three independent experiments. **p < 0.01, *p < 0.05.

As PD-L1, NO, IL-10 and TGF-β are key factors in MSCs-mediated inhibition, we co-cultured CD4^+^ T cells with OE-MSCs treated with or without IL-17, and then analyzed mRNA levels and protein levels of these suppressive factors in OE-MSCs or IL-17-treated OE-MSCs. In Figure 2A, mRNA levels of PD-L1, iNOS, IL-10 and TGF-β were reduced after OE-MSCs stimulated with IL-17, and the protein levels of NO, IL-10 and TGF-β in the culture supernatants were also significantly down-regulated in IL-17-stimulated OE-MSCs (Figure 2B).

### IL-17 inhibits the expansion of Tregs induced by OE-MSCs

**Figure 3:**
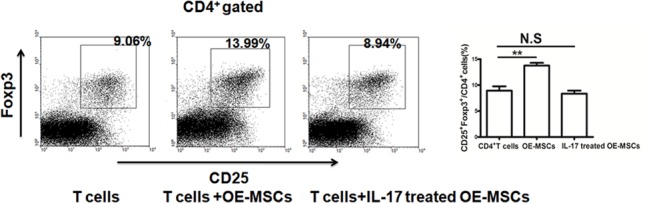
IL-17 reduces the capacity of OE-MSCs in inducing the expansion of Tregs OE-MSCs were treated with or without IL-17 (20 ng/ml) for 48 h, and then cells were harvested to co-culture with CD4^+^ T cells in the presence of anti-CD3 mAb and anti-CD28 mAb for 72 h, and then the proportion of CD4^+^CD25^+^Foxp3^+^ Treg cells in the co-culture system was analyzed by flow cytometry. Data are presented as mean ± SD pooled from three independent experiments. **p < 0.01, N.S represents no significance.

It is acknowledged that MSCs could promote the expansion of CD4^+^CD25^+^Foxp3^+^ Treg cells [12]. To observe the capacity of IL-17-treated OE-MSCs to induce the expansion of Tregs, we co-cultured OE-MSCs with CD4^+^ T cells. As shown in Figure 3, the percentage of CD4^+^CD25^+^Foxp3^+^ Treg cells was decreased in IL-17-treated OE-MSCs group when compared to the OE-MSCs group, which indicates that IL-17 could block the capacity of OE-MSCs in inducing the expansion of Tregs.

### IL-17R knockdown -OE-MSCs exhibits more efficient immunotherapeutic effect in CIA mice

**Figure 4:**
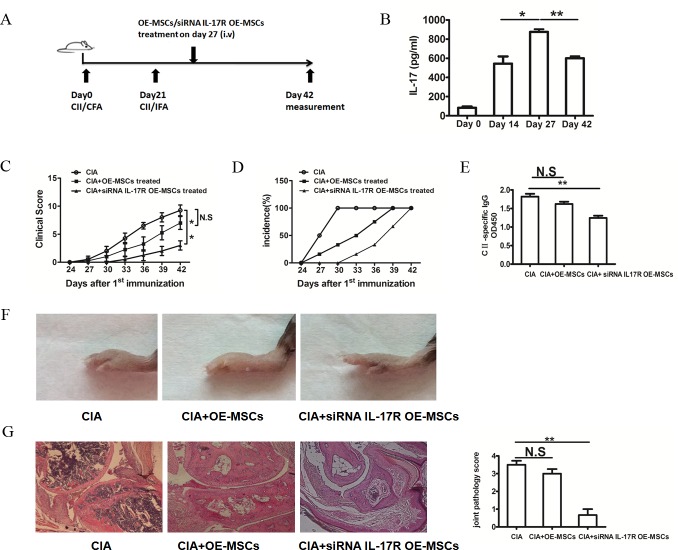
IL-17R knockdown OE-MSCs efficiently ameliorates the development of CIA **A.** Graphic scheme of CIA induction and OE-MSCs administration. DBA/1J mice were immunized with CII/CFA on day 0 and boosted with CII/IFA on day 21. Treatment groups were intravenously injected with OE-MSCs (1×10^6^) transfected with IL-17R siRNA or negative control on day 27 after CII/CFA immunization. Mice were then sacrificed on day 42. **B.** IL-17 levels on day 0, day 14, day 27 and day 42 during the CIA development were detected by ELISA. **C.**, **D.** Clinical score (C) and incidence of arthritis development (D) in immunized mice treated with OE-MSCs transfected with IL-17R siRNA or negative control were monitored every 3 days (n=6 per group). **E.** Serum levels of CII-specific autoantibodies from OE-MSCs transfected with IL-17R siRNA or negative control were detected by ELISA. **F.** Photos of hind paws from CIA mice treated with OE-MSCs transfected with IL-17R siRNA or negative control. **G.** Representative section of hind paws stained with hematoxylin and eosin, then assessed for histopathological scores of joint tissue from three groups. Results are expressed as mean ± SD. **p < 0.01,*p < 0.05, N.S represents no significance.

IL-17 has recently been implicated in the pathogenesis of multiple autoimmune diseases, including RA and the mouse model CIA. We found that IL-17 in the serum of CIA mice was markedly increased on day 14 after the first immunization, and reached its apogee on day 27 (Figure 4B). All mice developed long-lasting elevation of serum IL-17 even before they developed arthritis. Next, we sought to determine whether IL-17 could regulate the function of OE-MSCs and affect the therapeutic effect of OE-MSCs in the treatment on CIA. Small interference RNA (siRNA) was used to knock down the IL-17R level in OE-MSCs. Adoptive transfer of OE-MSCs or IL-17R siRNA treated OE-MSCs (siRNA IL-17R OE-MSCs) into CIA mice were performed on day 27 as the Figure 4A described. Then, the arthritis onset and disease progression were analyzed. As shown in Figure 4C and 4D, the clinical score was decreased and the development of arthritis was strikingly delayed in the siRNA IL-17R OE-MSCs group, whereas the OE-MSCs presented relative worse therapeutic effect under the environment with high concentration of IL-17. In addition, serum levels of anti-CII autoantibodies were significantly decreased in IL-17R siRNA treated OE-MSCs group than those in control groups, which was also slightly lower than OE-MSCs-treated mice (Figure 4E). Moreover, the swelling degree of the paws was significantly ameliorated in the siRNA IL-17R OE-MSCs treated mice (Figure 4F), and the histological analysis further showed significant improvement in the injury of joint (Figure 4G). Taken together, these data suggest that IL-17R siRNA treated OE-MSCs have more efficient effect on the treatment of CIA especially under the high level of IL-17 condition, which implies their great potential in RA treatment.

### IL-17R knockdown -OE-MSCs effectively reduce inflammatory responses in collagen-induced arthritis

**Figure 5:**
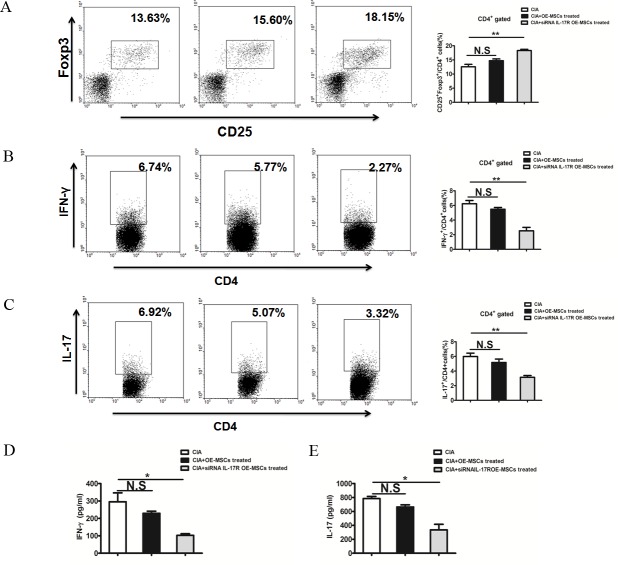
IL-17R knockdown OE-MSCs enhances Treg cells and reduces Th1 and Th17 cell responses in collagen-induced arthritis **A.**, **B.**, **C.** CD4^+^CD25^+^Foxp3^+^ Treg cells (A), CD4^+^IFN-γ^+^ Th1 (B) and CD4^+^ IL-17A^+^ Th17 (C) in the draining lymph nodes of CIA mice treated with OE-MSCs transfected with IL-17R siRNA or negative controls (*n* = 3/group). **D.**, **E.** Serum levels of IFN-γ (D) and IL-17 (E) in CIA mice in these three groups were measured by ELISA. Results are expressed as mean ± SD. **p < 0.01,*p < 0.05. N.S represents no significance.

The clinical amelioration and histological verification in CIA mice strongly suggest that IL-17R knockdown OE-MSCs are a potent tolerogenic agent that could suppress the autoimmune responses in CIA. We next investigated the mechanisms underlying the decrease in the severity of CIA. As shown in Figure 5A, the proportion of CD4^+^CD25^+^Foxp3^+^ Tregs in draining lymph nodes (dLN) was significantly increased after the IL-17R siRNA treated MSCs were injected in to the CIA mice, whereas the OE-MSCs treated group did not lead to the significant enhanced Tregs. Moreover, percentages of CD4^+^IFN-γ^+^Th1 cells (Figure 5B) and CD4^+^IL-17^+^Th17 cells (Figure 5C) were decreased after siRNA IL-17R OE-MSCs treatment. In addition, levels of proinflammatory cytokines IFN-γ and IL-17 were also down-regulated in the serum of CIA mice (Figure 5D, 5E). All these data indicate that during the treatment of CIA, OE-MSCs with IL-17R knocked down exhibit more efficient effect in reducing the inflammatory response by inducing the expansion of Tregs, thus reducing the Th1 and Th17 responses and relevant inflammatory factors as compared to the OE-MSCs, especially in the high level of IL-17 environment *in vivo*.

## DISCUSSION

Previous studies indicate that MSCs are increasingly being used to treat autoimmune and systemic inflammatory diseases [16, 17]. Although the detailed mechanism of MSC-based immune therapy has not been fully elucidated [18], bone marrow-derived MSCs might exert immunosuppressive properties by modulating T cells [19]. Many studies have demonstrated that MSCs can suppress the activation and function of various cells of the innate and adaptive immune systems, including by modulating T and B cell proliferation and differentiation, dendritic cell maturation as well as NK activity [20]. It is reported that MSCs could induce the generation of Tregs from CD4^+^ or CD8^+^ T cells, and these Tregs have powerful immunosuppression that strongly inhibit lymphocytes [21, 22]. Furthermore, MSCs have been also demonstrated to generate induced Tregs (iTregs) which could suppress effector T cells [23].

However, several recent reports showed that bone marrow-derived MSCs can be switched immunosuppression function by microbial molecular patterns through inflammatory cytokines [24]. MSCs can respond differently to different inflammatory stimuli [25]. MSCs acquire distinct immunophenotypes and activate different signaling pathways that may regulate immune responses. IL-17 is an inflammatory cytokine which is mainly produced by immune cells, such as Th17 and γδ T cells [26]. IL-17 is believed to play a critical role in orchestrating the inflammatory response in CIA [27, 28]. It was confirmed by reports showing that CIA was suppressed in IL-17-deficient mice and administration of neutralizing anti-IL-17 antibodies significantly reduced the severity of CIA [29]. Moreover, it has been demonstrated that serial serum IL-17 levels is increased after collagen immunization in CIA mice [27]. Therefore, our aim of this study was to investigate the effect of the inflammatory cytokine IL-17 on OE-MSCs, and then further explore the therapeutic effect of OE-MSCs on the rheumatoid arthritis under the environment with high level of IL-17.

Here we firstly showed that IL-17 could decrease the inhibitory capacity of OE-MSCs in suppressing effector T cell proliferation. Further investigation demonstrated that IL-17 decreased the suppressive ability of OE-MSCs mainly by down-regulating the levels of inhibitory factors produced by OE-MSCs, including soluble factors NO, IL-10, TGF-β as well as cell surface-expressed inhibitory molecule PD-L1. It has been reported that the downstream signaling of IL-17 involves several pathways, including NFκB, MAPK (p38/JNK/ERK) and C/EBP [30]. In addition, Yang *et al* have found that IL-17 produced by a subset of IL-17^+^ MSCs could down-regulate the level of TGF-β in MSCs *via* NFκB pathway [31]. Nevertheless, further studies are warranted to elucidate the molecular mechanism underlying the regulatory effect of IL-17 on the suppressive ability of OE-MSCs.

Previous studies using bone marrow-derived MSCs in CIA model have generated conflicting results [32, 33]. Although more and more studies have shown that MSCs are “licensed” to exert their immunomodulatory effects after stimulation with IFN-γ in the presence of one or more other cytokine(s), including TNF, IL-1α or IL-1β, some other interesting studies also showed the opposite effect of MSCs after inflammatory stimulation [34, 35]. For instance, MSCs can provide a pro-inflammatory signature by the Toll-like receptor 4 activation [25]. These findings bring new insight to understanding of the crosstalk between MSCs and the inflammatory niche and provide practical information for improving the therapeutic potential of MSCs in the treatment of autoimmune diseases. Although IL-17 production has been demonstrated to increase in CIA, it was not known whether IL-17 had any effect on OE-MSCs during the treatment of CIA. Our studies have implicated that IL-17 has a profound influence in the treatment effect of OE-MSCs in CIA model. Compared with OE-MSCs, OE-MSCs interfered with IL-17R exerted more potent anti-proliferative activity and induced Treg expansion and decrease Th1 and Th17 cell generation *in vivo*, which might be attributed to the recovery of their capacity to produce high levels of IL-10, TGF-β and NO.

In summary, our study has demonstrated that IL-17 obviously reversed the immunosuppressive function of OE-MSCs. Important for their clinical transformation is the fact that OE-MSCs maintain the immunosuppressive activity observed *in vitro* after their infusion *in vivo*. In our findings, therapy with siRNA IL-17R OE-MSCs has a highly beneficial method for specifically suppressing the immune response in CIA model. We believe that these results could help shape future clinical strategies for the treatment of autoimmune diseases.

## MATERIALS AND METHODS

### Isolation and culture of OE-MSCs

OE-MSCs were obtained from the nasal cavity by delicately discarding the turbinates and then cut the retained olfactory epithelium into small pieces and culture in flasks with the medium (Dulbecco's modified Eagle's medium (DMEM)/HAM'sF-12 supplemented with 15% fetal calf serum) (Gibco, Carlsbad, CA). One week later, non-adherent cells were removed and stem cells will begin to invade the culture flasks. After reached 80% confluency in the dish, adherent cells were trypsinized (0.05% trypsin-ethylene-diaminetetraacetic acid at 37°C for 2 min) and expanded for 3 passages.

### Osteogenic and adipogenic differentiation of OE-MSCs

To assess the multipotentiality of OE-MSCs, cells were induced by osteogenic medium and adipogenic medium *in vitro*. The osteogenic induction medium was a mixture of MEM-supplemented with 0.1μM dexamethasone (SigmaAldrich, St.Louis, MO), 10 mM β-glycerol phosphate (SigmaAldrich, St.Louis, MO) and 0.2 mM ascorbic acid (SigmaAldrich, St. Louis, MO). After 21 days, cells were subjected to alizarin red staining (Cyagen, Guangzhou, China). To induce adipogenic differentiation, OE-MSCs were cultured in mesenchymal stem cell adipogenic differentiation medium (Cyagen, Guangzhou, China). 14 days later, cells were subjected to oil red O staining (Cyagen, Guangzhou, China).

### Mice

DBA1/J mice (8-10 weeks, male) were obtained from the Shanghai Laboratory Animal Center (Shanghai, China) and maintained in the Jiangsu University Animal Center (Jiangsu, China). All animal experiments were approved by the Jiangsu University Animal Ethics and Experimentation Committee.

### Induction and assessment of arthritis

For CIA induction, we immunized male DBA/1J mice by injecting 100 μg of bovine type II collagen (Chondrex, WA, USA) emulsified with complete Freund's adjuvant (C II:CFA=1:1, CFA: Sigma Aldrich, St. Louis, MO) *via* the base of the tail. 21 days later, a booster emulsion prepared by a boost with 100 μg of the same bovine collagen type II emulsified in Freund's incomplete adjuvant (Sigma Aldrich, St.Louis, MO) near the primary injection site. To determine the effect of OE-MSCs treatment, mice were received a single intravenous injection of 1×10^6^ OE-MSCs or OE-MSCs treated with IL-17R siRNA on day 24 after 1st immunization. From day 21, mice were scored for signs of arthritis every 3 days. Each paw was evaluated and scored individually using a 0 to 4 scoring system. The paw scores were summed to yield the clinical score, with a maximum score of 16 for each mouse. Each paw score was judged as follows: 0, no evidence of erythema and swelling; 1, erythema and mild swelling confined to the tarsals or ankle joint; 2, erythema and mild swelling extending from the ankle to the tarsals; 3, erythema and moderate swelling extending from the ankle to metatarsal joints; and 4, erythema and severe swelling encompass the ankle, foot and digits, or ankylosis of the limb [36]. The assessment was performed by 2 of the authors who were blinded to the identity of the specimens.

### Histopathologic analysis of the joints

After mice were sacrificed on day 42 after the first immunization, murine joint tissue specimens were obtained and fixed in 10% phosphate-buffered formalin for 3 days. Tissue sections (4μm thickness) were stained with hematoxylin and eosin to examine morphologic features and assess the histologic arthritis score. The extent of synovitis, pannus formation, and bone/cartilage destruction was determined using a graded scale as follows: grade 0, no signs of inflammation; 1, mild inflammation with hyperplasia of the synovial lining without cartilage destruction; 2, pannus formation and cartilage erosion; 3, major erosion of cartilage and subchondral bone; 4, loss of joint integrity and ankylosis [37, 38]. The assessment was performed by 2 of the authors who were blinded to the identity of the specimens.

### Co-culture of OE-MSCs and T cells

OE-MSCs were stimulated with or without IL-17 (0, 10, 20, 50 ng/ml) for 48 h, and then cells were harvested to co-cultured with CD4^+^ T cells that were sorted from wild-type mice using CD4^+^ T cells microbeads (Miltenyi Biotec, Bergisch Gladbach, Germany) in the presence of anti-CD3 mAb (10mg/mL, Biolegend, SanDiego, CA) and anti-CD28 mAb (5mg/mL, Biolegend, San Diego, CA) for 72 h and then cells were pulsed with [^3^H]-thymidine (Pharmacia, Stockholm, Sweden, 1μCi/well) for the last 16 h of culture.

### Flow cytometric analysis

To identify the OE-MSCs phenotype, single cell suspensions were stained with relevant fluorochrome-conjugated mouse CD29, CD90, CD44, CD45, CD11b IL-17R mAbs (eBioscience, San Diego, CA). For detection of regulatory T (Treg) cells, anti-CD4, anti-CD25, and anti-Foxp3 mAbs (eBioscience, San Diego, CA) were performed using Foxp3 staining buffer set (eBioscience, San Diego, CA). For intracellular cytokine staining, single cell suspensions were stimulated with phorbol myristateacetate (50ng/ml, SigmaAldrich, St.Louis, MO), ionomycin(1μg/ml, Enzo, Farmingdale, NY) and monensin (2 μg/ml, Enzo, Farmingdale, NY) at 37°C and in a 5% CO_2_ atmosphere for 5 hours. Then cells were stained with anti-CD4 mAbs (eBioscience, San Diego, CA), fixed, permeabilized and stained with anti-IFN-γ mAb or anti-IL-17 mAb (eBioscience, San Diego, CA). As controls, the appropriate isotype-matched antibodies were used for each staining. Flow cytometry was performed using a FACS Calibur flow cytometer (eBioscience, San Diego, CA), and the data was analyzed by WinMDI 2.8 software.

### Measurement of autoantibodies and cytokines

Serum levels of type II collagen-specific antibody were measured by enzyme-linked immunosorbentassay (ELISA) as previously described. Levels of IL-17, TGF-β, IFN-γ, IL-10 in culture supernatants or serum of murine samples were measured using ELISA Ready-SET-Go Kits (eBioscience, San Diego, CA) following the manufacturer's protocol. The amount of NO was assessed by determining the concentration of nitrite accumulated in culture supernatants using the colorimetric Griess reaction (Promega, Madison, WI).

### Quantitative real-time PCR (qRT-PCR)

To determine gene expression, total RNA was extracted with TRIzol reagent (Invitrogen). Isolated total RNA was reversed-transcribed with ReverTra Ace qPCR RT kit (TOYOBO) according to the manufacturer's instructions. The reverse transcript PCR and qRT-PCR were performed as described previously [39]. The sequences for the primers used are: IL-17R, 5-AGTGTTTCCTCTACCCAGCAC-3 (forward), 5-GAAAACCGCCACCGCTTAC-3 (reverse); TGF-β, 5-AACCGGCCCTTCCTGCTCCTCAT-3 (forward), 5-CGCCCGGGTTGTGTTGGTTGTAGA-3 (reverse); IL-10, 5-GGTTGCCAAGCCTTATCGGA-3(forward), 5-ACCTGCTCCACTGCCTTGCT-3 (reverse); β-actin, 5-TGGAATCCTGTGGCATCCATGAAAC-3 (forward), 5-TAAAACGCAGCTCAGTAACAGTCCG-3 (reverse); iNOS, 5-GAGCCCTCAGCAGCATCCAT-3 (forward), 5-GGTGAGGGCTTGGCTGAGTG-3 (reverse); 18s, 5-TCCGGAGAGGGAGCCTGAGA-3 (forward), 5-GCACCAGACTTGCCCTCCAA-3 (reverse); PD-L1, 5-CTGCTTGCGTTAGTGGTGT-3 (forward), 5-TGGTTGATTTTGCGGTATG-3 (reverse). Relative quantification of mRNA expression was calculated by the comparative threshold cycle (Ct) method.

### Transfection

IL-17R siRNA was designed and synthesized by RiboBio Co. Ltd. Nonspecific scramble siRNA was used as negative control. OE-MSCs were transfected with IL-17R siRNA or negative control using Entranster-R (Engreen Biosystem) according to the manufacturers' instructions [40]. The final concentration of oligonucleotides was 100 nM.

### Statistics

The statistical significance of differences between groups was determined by the Student's t test and one-way analysis of variance. All analyses were performed using SPSS16.0 software. Differences were considered significant at a *p* level less than 0.05.

## SUPPLEMENTARY MATERIAL


