# Supplementary material for: IL-17 down-regulates the immunosuppressive capacity of olfactory ecto-mesenchymal stem cells in murine collagen-induced arthritis
Source: Oncotarget. 2016 Jun 23;7(28):42953–62. doi: 10.18632/oncotarget.10261 (PMC5189999; doi:10.18632/oncotarget.10261)
Supplement: Supplementary file 1 [file oncotarget-07-42953-s001.pdf]

IL-17 down-regulates the immunosuppressive capacity of olfactory ecto-mesenchymal stem cells in collagen-induced arthritis

Supplementary Material

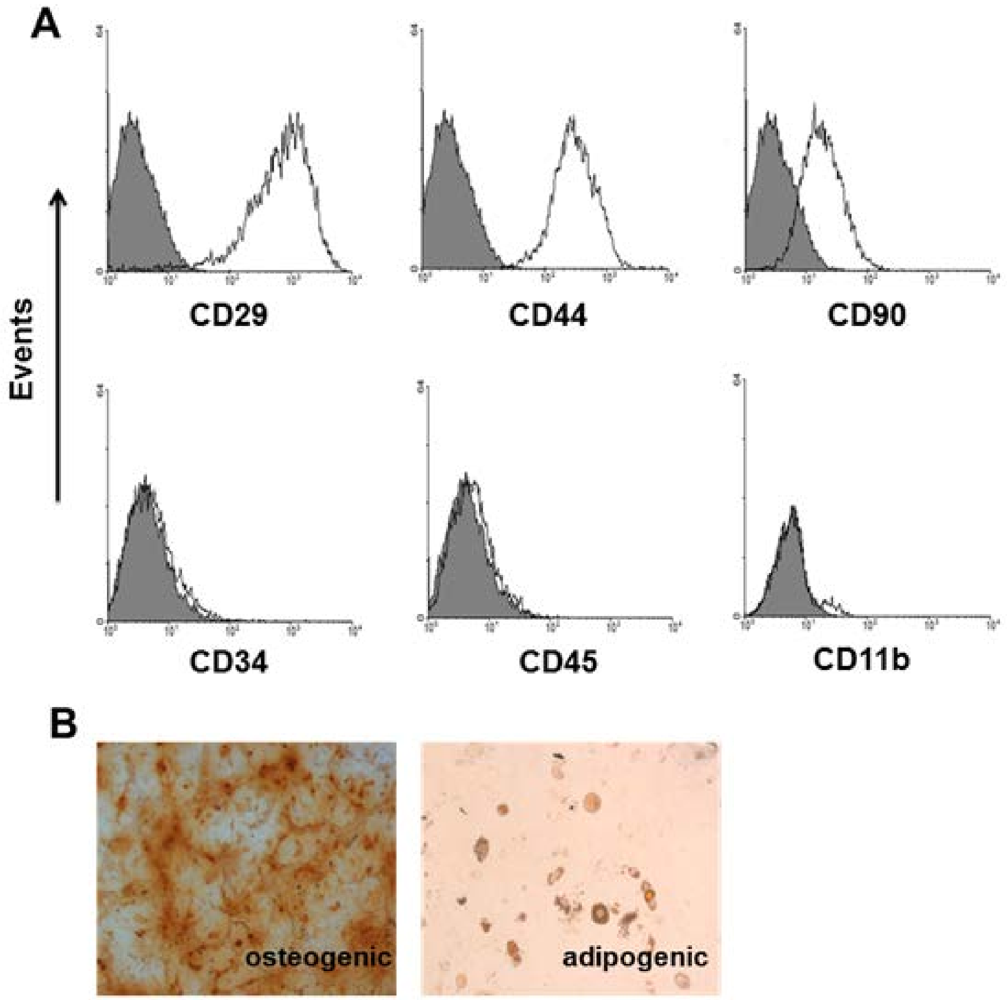

Supplementary Figure S1: Phenotypic and functional characterization of OE-MSCs. A. Flow cytometric analysis of surface-marker expression on OE-MSCs, including representative MSC markers CD29, CD44 and CD90, hematopoietic cell markers CD34 and CD45 and myeloid cell marker CD11b. **B.** OE-MSCs were

cultured in osteogenic/adipocytes conditions for 14/21 days, followed by alizarin red staining (left panel) or oil red O (right panel), respectively (original magnification × 10). Results are representative of three independent experiments.
